# Communities of endophytic fungi in a Puerto Rican rainforest vary along a gradient of disturbance due to Hurricane Maria

**DOI:** 10.1002/ece3.9618

**Published:** 2022-12-14

**Authors:** Nicole Colón Carrión, Chad Lozada Troche, A. Elizabeth Arnold

**Affiliations:** ^1^ School of Plant Sciences University of Arizona Tucson Arizona USA; ^2^ Department of Biology University of Puerto Rico Cayey Puerto Rico; ^3^ Department of Ecology and Evolutionary Biology University of Arizona Tucson Arizona USA

**Keywords:** Ascomycota, biodiversity, climate change, intermediate disturbance hypothesis, plant‐fungal symbioses, tropical forest

## Abstract

Increases in the frequency and intensity of hurricanes influence the structure, function, and resilience of Caribbean forests. Trees in such forests harbor diverse fungal endophytes within leaves and roots. Fungal endophytes often are important for plant health and stress responses, but how their communities are impacted by hurricanes is not well known. We measured forest disturbance in Carite State Forest in Puerto Rico ca. 16 months after the passage of Hurricane Maria, a Category 4 storm. In three sites, each comprising three plots representing a local gradient of hurricane disturbance, we evaluated soil chemistry and used culture‐free analyses to measure richness, phylogenetic diversity, and composition of endophyte communities in leaves and roots. We found that endophyte richness did not vary significantly among plant families or as a function of soil chemistry. Instead, leaf endophytes peaked in richness and decreased in phylogenetic diversity at intermediate levels of disturbance. Root endophytes did not show such variation, but both leaf‐ and root endophyte communities differed in species composition as a function of disturbance across the forest. Locations with less disturbance typically hosted distinctive assemblages of foliar endophytes, whereas more disturbed locations had more regionally homogeneous endophyte communities. Together, our results show that changes in endophyte richness and phylogenetic diversity can be detected in aboveground tissues more than a year after major storms. In turn, pervasive shifts in endophyte community composition both aboveground and belowground suggest a subtle and lasting effect of hurricanes that merits further study, potentially contributing to the promotion of spatially heterogeneous endophyte assemblages at a landscape scale in these diverse island forests.

## Graphical abstract

Even more than a year after the catastrophic passage of Hurricane Maria, communities of endophytic symbionts in a Puerto Rican rainforest bear a signature of forest disturbance.

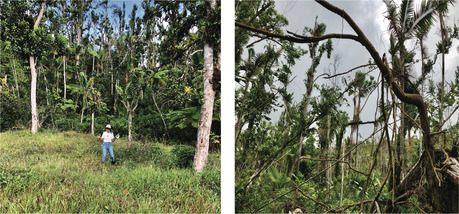

## Resumen

Los aumentos en la frecuencia e intensidad de los huracanes influyen en la estructura, función, y resiliencia de los bosques del Caribe. Los árboles en dichos bosques tienen diversos hongos endófitos dentro de las hojas y raíces. Los hongos endófitos pueden ser importantes para la salud de las plantas y las respuestas a el estrés, pero no se conoce bien cómo los huracanes afectan a sus comunidades. Medimos la perturbación forestal en el Bosque Estatal de Carite en Puerto Rico a 16 meses después del paso del Huracán María (huracán categoría 4). En tres sitios, cada uno de los cuales incluyeron tres parcelas que representan un gradiente local de perturbación debido al huracán, evaluamos la química del suelo y utilizamos análisis libres de cultivos para medir la riqueza, la diversidad filogenética y la composición de las comunidades de endófitos en las hojas y raíces de las plantas. Encontramos que la riqueza de endófitos no varió significativamente entre las familias de plantas o en función de la química del suelo. Los endófitos de hojas alcanzaron su punto máximo en riqueza y disminuyeron en diversidad filogenética en niveles intermedios de perturbación forestal. Los endófitos de raíces no mostraron tal variación, pero ambas comunidades de endófitos difirieron en la composición de especies en función de la perturbación en el bosque. Las ubicaciones con menos perturbaciones generalmente tenían conjuntos distintivos de endófitos foliares, mientras que las ubicaciones más perturbadas tenían comunidades de endófitos regionalmente más homogéneas. Nuestros resultados muestran que los cambios en la riqueza de endófitos y la diversidad filogenética se pueden detectar en los tejidos de las hojas más de un año después de grandes tormentas. A su vez, los cambios generalizados en la composición de la comunidad de endófitos de hojas y raíces sugieren un efecto sutil y duradero de los huracanes que amerita un mayor estudio, lo que podría contribuir a la promoción de ensamblajes de comunidades de endófitos heterogéneas en diversos bosques.

## 1 INTRODUCTION

Hurricanes are the most high‐energy events that affect the Caribbean (Alaka, 1976; Boucher, 1990; Brokaw & Grear, 1991), profoundly impacting ecosystem structure and function in forests of the region (Brokaw & Walker, 1991; Tanner et al., 1991). On September 20, 2017, Hurricane Maria struck Puerto Rico as a Category 4 storm with sustained winds up to 249.5 km/h and heavy rainfall (López‐Marrero et al., 2019; Pasch et al., 2018; Wachnicka et al., 2020). As the strongest hurricane in Puerto Rico in nearly 90 years, Maria's wind gusts, rainfall, and landslides damaged mature forests in many parts of the island, with an estimated 23–31 million trees severely damaged or killed (Feng et al., 2018; see also Uriarte et al., 2019). Accordingly, comparisons of pre‐hurricane (2015 and 2016) and post‐hurricane surveys revealed reductions in vegetation cover after Hurricane Maria's passage (Hosannah et al., 2020; Hu & Smith, 2018; Van Beusekom et al., 2018).

Hurricane‐driven tree mortality and shifts in forest structure can impact plant communities directly and thus alter ecosystem services immediately and visibly (Brokaw & Grear, 1991; Lugo, 2008; Paudel & Battaglia, 2021; Xi, 2015). However, hurricanes also may have less visible, long‐term effects if symbionts that are important for forest trees are impacted negatively by such storms (see Cantrell et al., 2014; Miller & Lodge, 1997; Shiels et al., 2014). In addition to mycorrhizal fungi, forest trees in Puerto Rico host diverse communities of fungal endophytes in healthy tissues such as leaves and roots (e.g., Bayman et al., 1997, 1998; Gamboa & Bayman, 2001; Lodge et al., 1996; Maillard et al., 2020). Some endophytes can improve the resilience of plants against herbivores, pathogens, and abiotic stressors (see Arnold et al., 2003; Bouzouina et al., 2021; Czarnoleski et al., 2013; Giauque et al., 2019; Moghaddam et al., 2021), influencing nutrient cycling, plant–soil feedbacks, and plant growth (Bai et al., 2019; Dhyani et al., 2019; Dong et al., 2021; Korkama‐Rajala et al., 2008). For these reasons, endophytes are increasingly considered important for forest health (Arnold et al., 2003; Bamisile et al., 2018; Christian et al., 2019; Griffin & Carson, 2018; Rodriguez et al., 2009; Yamaji et al., 2016), and understanding their responses to disturbances such as those caused by hurricanes is important for understanding the dynamics of forest regeneration (see Busby et al., 2022).

Effects of hurricanes on soilborne microbial communities in forests have been studied previously (e.g., Cantrell et al., 2014; Eaton et al., 2020), providing some insight into the ways in which fungi associated with plants may respond to forest disturbance. For example, soil microbes and fungi shift in community composition in response to disturbance by hurricanes, typically decreasing in diversity due to dominance by a smaller number of species (Cantrell et al., 2014; Eaton et al., 2020; Lodge, 1997). Soilborne microbial biomass also changes in response to forest damage, reflecting an increase in C and N concentrations in soil and altered soil processes (Lodge et al., 2016; Reed et al., 2020).

In comparison, relatively little is known about how forest disturbance due to hurricanes may impact endophyte communities. Endophytes of forest plants typically are transmitted via air spora (leaves) and infection from soil (roots). Thus, the endophytes present in a tropical forest tree represent a subset of regionally available species, with strong impacts from ultraviolet radiation and other environmental factors that often are changed in disturbed forests (Oita et al., 2021). Because such endophytes typically have life stages outside of living tissues (see Herre et al., 2007; Nelson et al., 2020; Thomas et al., 2016), they may be affected by environmental shifts much like soil‐inhabiting fungi. However, indirect effects of hurricanes on endophytes also may occur: hurricanes typically open forest canopies and increase litter accumulation, resulting in higher temperatures, changes in the light regime, and shifts in nutrient availability in the understory (Shiels et al., 2010) that impact plant physiology (Wen et al., 2008). Thus, forest disturbance due to hurricanes may select for: (1) endophyte species that are well adapted to withstand abiotic stress and/or a dynamic environment; (2) species that are more likely to have pathogenic or saprotrophic life stages, capitalizing on plant damage; and/or (3) specialized functional groups that facilitate the growth of host plants, ultimately benefiting the endophytes themselves (see Kandalepas, 2012; Maillard et al., 2020; see also Busby et al., 2022). If the patchy disturbance regimes imposed by hurricanes lead to different environmental pressures in different areas (see Kandalepas, 2012), such storms may lead to small‐scale variation in communities at landscape scales (Gannon & Martin, 2014), ultimately shaping the distribution and subsequent impact of endophytes forest‐wide.

The goal of this study was to understand how disturbance due to hurricanes may impact endophyte communities in forest plants. Because impacts of hurricanes vary in intensity even within the same region (e.g., Lugo, 2000), it is possible to quantify effects of hurricanes on endophytes without confounding effects of large geographic distances or major shifts in edaphic or climate conditions. Here, we measured disturbance due to Hurricane Maria in Carite State Forest of Puerto Rico ca. 16 months after the storm's passage, when permits and safe access were allowed by forest managers. In three sites, each comprised of three plots that represented a local gradient of disturbance due to the hurricane, we evaluated soil chemistry and collected leaves and roots, which we used in culture‐free analyses to measure the richness, phylogenetic diversity, and composition of endophyte assemblages. We considered it plausible that these aspects of endophyte communities would vary linearly with the severity of forest damage, but also realized that relationships between endophyte richness or phylogenetic diversity and forest damage might not be linear, instead peaking or decreasing markedly only at intermediate levels of disturbance. Although often debated for its theoretical basis and empirical insights (e.g., see Fox, 2013a, 2013b; Sheil & Burslem, 2013; see also Wang & Lin, 2019), the typical framework for considering such patterns is the intermediate disturbance hypothesis (IDH; Connell, 1978), which provides an expectation that richness or diversity is maximized when ecological disturbances are moderate.

Under this framework, we predicted that species richness of endophytes would be greatest where hurricane disturbances were intermediate (prediction 1). We also expected that endophytes in low‐ and high‐disturbance sites might represent a broad phylogenetic diversity, with the former representing diverse, mature communities and the latter representing early successional communities. Therefore, we predicted that phylogenetic diversity would decrease in intermediately disturbed areas relative to less‐ or more severely disturbed areas (prediction 2). Based on previous research showing that endophyte communities can vary spatially over small distances in neotropical forests (e.g., Higgins et al., 2014), we anticipated that endophyte communities would differ spatially but also as a function of forest disturbance (prediction 3). Finally, we predicted that areas with more extensive disturbance would have relatively similar endophyte communities to one another, whereas areas with less damage would have locally distinctive endophyte communities (prediction 4).

## 2 METHODS

### 2.1 Site characterization

This study was conducted at Carite State Forest near Cayey, Guayama, and Patillas in southeastern Puerto Rico (18°3′56.8″ N, 66°6′46.3″ W). The forest ranges from 250 to 903 m above sea level (m a.s.l.) with an approximate area of 2428 ha. The mean annual rainfall is 2159 mm and the mean annual temperature is 22°C (DRNA, 2008). Three life zones occur in the area: humid subtropical forest (0.9%), very humid subtropical forest (98.6%), and very humid montane low forest (0.5%; DRNA, 2008).

**FIGURE 1:**
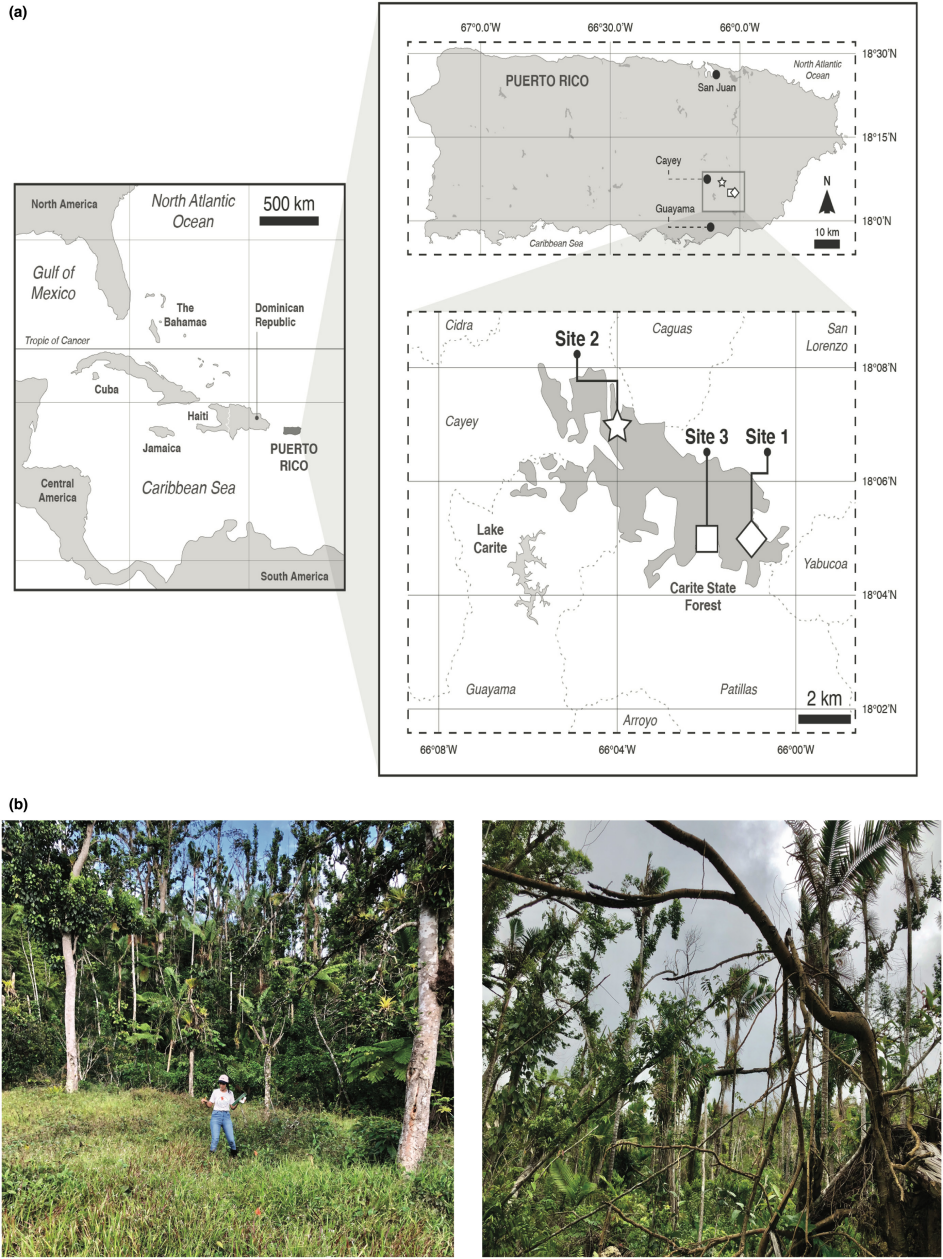
(a) Carite State Forest, Puerto Rico. Each site included three plots representing a local gradient of forest damage scores, which were used to estimate disturbance due to Hurricane Maria. (b) Representative damage caused by Hurricane Maria at Carite State Forest. Left: plot with a low damage score, viewed from beyond the forest edge (site 1, plot 1). Right: plot with a high damage score (site 3, plot 3).

We selected three sites in humid and very humid subtropical forest ranging from 560 to 655 m a.s.l.: two areas at Charco Azul Recreational Area, and one at Guavate Recreational Area (Figure 1a). The sites were similar in terms of forest structure and land use history prior to Hurricane Maria (H. Serrano, pers. comm.).

In each site, we characterized disturbance caused by Hurricane Maria following Basnet et al. (1992) with minor modifications. Briefly, in December 2018 we established three 4 × 5 m plots in each site. Each plot was centered on a canopy tree chosen randomly, with at least 100 m between plots. At each of the three plots per site, we established four 20 m line transects in cardinal directions from the focal tree. Along each transect, trees ≥1 cm diameter at breast height (DBH) were classified in the following mutually exclusive categories: defoliated but standing (DF), uprooted (UT), broken branches (BB), and broken stems (BS; Basnet et al., 1992). We summed the values obtained for each category to obtain a forest damage score for each plot, which we log‐transformed prior to analysis and considered representative of the relative disturbance in each site that could be ascribed to Hurricane Maria. Damage scores at the nine study plots ranged from 1 to 30 (Table 1).

**TABLE 1:** Site characteristics, forest damage scores for characterizing disturbance, and soil chemistry data collected at plots in Carite State Forest, Puerto Rico, 16 months after the passage of Hurricane Maria.

Site	Plot	Lat (N)	Long (W)	Elevation (m a.s.l.)	Damage score	Soil chemistry (PPM)		
						NO_3_^−^	P	K
1	1	18°05′25.52″	66°01′57.41″	560.8	4	29.33	1.60	189.67
1	2	18°05′26.05″	66°01′57.41″	560.8	6	27.33	3.10	275.00
1	3	18°05′25.33″	66°01′58.14″	560.8	8	38.00	1.02	103.67
2	1	18°07′21.60″	66°04′11.38″	606.5	1	30.33	1.55	191.67
2	2	18°07′19.53″	66°04′10.05″	606.5	10	37.00	ND	101.00
2	3	18°07′19.32″	66°04′11.04″	606.5	12	29.33	2.88	275.67
3	1	18°05′28.77″	66°02′03.77″	655.3	11	31.33	1.23	294.33
3	2	18°05′29.40″	66°02′04.54″	618.7	21	35.33	1.02	210.00
3	3	18°05′27.84″	66°02′04.32″	618.7	30	43.33	0.43	204.67

*Note*: Plant taxa collected in each plot are shown in Table S4.

Abbreviations: m a.s.l., meters above sea level; ND, not determined.

In each plot we selected five representative plants. From each plant we collected 10 leaves and three root cores (6.3 cm diameter and ~10 cm depth, collected beneath the edge of the canopy of each individual). Root identity was confirmed by tracing roots where possible, and/or matching DNA sequences from leaves and roots as described below. We measured stem richness and density, canopy height, diameter at breast height (DBH; ≥1 cm) of the focal tree, and canopy cover in each plot as in Oita et al. (2021) (Table S1). For soil chemistry analyses, we collected three soil cores (6.3 cm diameter) from three randomly chosen corners of each plot after leaf litter was cleared. They were pooled by plot and sent to the Puerto Rico Tropical Agriculture Research Station (TARS) for quantification of nitrate, available phosphorus and potassium.

### 2.2 Sample preparation

Soil samples were sieved to extract roots following Mukerji et al. (2002). Healthy leaves and roots collected from each individual plant were processed separately. In each case, tissues were cut into 1 cm‐segments, rinsed in running tap water for 60 s, and surface‐sterilized by agitation in 95% ethanol (30 s), 0.5% NaOCl^−^ (2 min), and 70% ethanol (2 min; Arnold & Lutzoni, 2007). We randomly selected three segments from each sample and stored them in an Eppendorf tube containing 1 ml of cetyl trimethylammonium bromide (CTAB) buffer. This was repeated three times per individual plant, such that our final sampling included 9 cm^2^ of leaf tissue and approximately 9 cm of root tissue per individual. Samples in CTAB were stored at −20°C prior to DNA extraction.

### 2.3 DNA extraction, amplification, and sequencing for endophytes

We extracted total genomic DNA from leaves and roots with the Qiagen DNeasy Plant Mini Kit (Qiagen) following U'Ren and Arnold (2017a). Each CTAB tube represented a single DNA extraction, such that each individual was represented by three DNA extractions from leaf tissue, and three DNA extractions from root tissue. DNA extractions were pooled by tissue type into a single leaf‐ or root sample for each individual following U'Ren and Arnold (2017a).

We amplified the fungal ribosomal internal transcribed spacer (ITS) region (i.e., ITS1, 5.8 S, and ITS2; U'Ren & Arnold, 2017a) via the polymerase chain reaction (PCR) in a two‐step process. The first PCR (PCR1) amplified the ITS region with primers ITS1F and ITS4, which were amended with universal sequences CS1 and CS2 (Integrated DA Technologies Inc.). Reactions were carried out in triplicate per pooled DNA sample, each with a total volume of 20 μl (10 μl of Phusion Flash Master Mix [Thermo Scientific]; 0.2 μl of 0.5 μM of each primer; 1.0 μl of 1 mg/ml of BSA [New England Biolabs]; 3.6 μl of molecular grade water [Fisher Scientific]; and 5 μl of DNA template). PCR1 was run on an MJ Research PTC‐200 Gradient Thermal Cycler® (Marshall Scientific) with the following parameters: 10 s at 98°C; 28 cycles of 1 s denaturation at 98°C, 5 s annealing at 57°C, and 20 s extension at 72°C; and a final extension of 1 min at 72°C. PCR products were stained with SYBR green and verified on a 2% agarose gel under ultraviolet (UV) light. The three PCR products per sample were pooled and diluted with molecular grade water based on band intensity (U'Ren & Arnold, 2017a). Negative controls (water as template) and extraction blanks were treated as above for PCR1, and their products were processed in the second PCR (PCR2), in parallel with our samples.

In PCR2, we added adapters and unique barcodes to amplicons from PCR1 (see Oita et al., 2021; Sarmiento et al., 2017; U'Ren et al., 2019). Amplifications were carried out as above with a total volume of 20 μl (10 μl of Phusion Flash Master Mix, 0.75 μl of 0.075 μM of each primer, 0.24 μl of 0.24 mg/ml of BSA, 8.01 μl of molecular grade water, and 1 μl of DNA template). The cycling parameters were 10 s at 98°C; seven cycles of 1 s denaturation at 98°C, 5 s annealing at 55°C, and 20 s extension at 72°C; and a final extension of 1 min at 72°C. Products were stained with SYBR green, verified with a 2% agarose gel under UV light, and sent to the University of Arizona Genetics Core (UAGC) for DNA quantification with a Bioanalyzer 2100 (Agilent Technologies). Products were pooled into a 2‐ml microcentrifuge tube to a final concentration of 20 ng of DNA (U'Ren & Arnold, 2017b) and shipped on dry ice for amplicon sequencing (Illumina MiSeq) at the University of Idaho IBEST Genomics Resources Core.

Two mock communities were included in our sequencing to identify primer bias, determine quality control parameters, and assess the correlation of operon number and read count (see Bowman & Arnold, 2021; Daru et al., 2018; Oita et al., 2021). The first community consisted of DNA from 31 phylogenetically diverse fungal taxa, represented in equal concentrations (“even” mock community; Daru et al., 2018; Table S2). The second mock community consisted of tiered DNA concentrations of the same fungal taxa (Daru et al., 2018; Table S2). Mock communities were treated as above and included with the samples for sequencing. We found that the even mock community produced unusual results consistent with contamination and degradation of DNA stocks, whereas the tiered mock community samples were effective positive controls. We therefore focused only on the tiered mock community as our positive control, as described below.

### 2.4 Plant identification

Plants were identified based on Axelrod (2011), Little et al. (1974), the database of the Institute for Regional Conservation (https://www.regionalconservation.org/index.html, with reference to Carite State Forest), and digitized specimens available from the Missouri Botanic Garden (MO; www.tropicos.org) and the University of Puerto Rico‐Rio Piedras (http://herbario.uprrp.edu). DNA from leaves was used to identify plants in cases in which morphological determination was not possible due to degradation of tissue following collection, when limited facilities were available for processing specimens. We amplified and sequenced the plant nuclear ribosomal ITS region because it was straightforward to amplify, but due to limitations in resolution we used ITS data to assign plants to the genus or family level only. PCR reaction volumes were 20 μl, including 10 μl of REDExtract‐N‐Amp PCR Reaction Mix (Sigma‐Aldrich), 0.8 μl of each 10 μM primer (ITS5/ITS4; see White et al., 1990), 7.4 μl of molecular biology grade water (Fisher Scientific), and 1 μl of DNA template (Table S3). The cycling parameters were 3 min at 94°C, 36 cycles of 30 s denaturation at 94°C, 30 s annealing at 54°C, and 1 min extension at 72°C; and a final extension of 10 min at 72°C (Hoffman et al., 2008). PCR amplifications were verified by electrophoresis as described above. Amplicons were treated with 1 μl ExoSAP‐IT (Affymetrix) and incubated for 60 min at 37°C and 15 min at 80°C (Bell, 2008) prior to bidirectional Sanger sequencing at the University of Arizona Genetics Core. Sequencing reactions included 5 μM of the PCR primers and were processed on a 373*xl* DNA Sequencer (Applied Biosystems). Contigs were assembled and edited with phred and phrap as implemented in Mesquite v.2.75 and verified by eye in Sequencher v.4.5 (Ewing & Green, 1998; Maddison & Maddison, 2017). Sequence data were compared via BLASTn to plant records in GenBank and cross‐referenced with the herbarium and field guides mentioned above (Altschul et al., 1990; Table S4).

Samples that failed to be identified were amplified with alternate primer pairs that also target the plant nuclear ribosomal ITS region (Table S3) in a total volume of 20 μl (10 μl of DreamTaq Hot Start Green Master Mix [Fisher Scientific], 0.8 μl of each 10 μM primer [ITS‐P3/ITS‐U4 or ITS‐P3/ITS‐U4/ITS‐P5], 6.4 μl of molecular biology grade water [Fisher Scientific], and 2 μl of DNA template; Cheng et al., 2016). Samples were amplified, sequenced, and identified as stated above.

DNA vouchers are retained at the University of Arizona. Representative sequence data have been submitted to GenBank (accessions OP390837‐OP390867).

### 2.5 Illumina sequence editing and quality control

Illumina reads were demultiplexed at the IBEST Genomics Core. Fast QC, Multi‐QC, and fastq_eestats2 command in USEARCH were used to assess read quality and determine maxEE and cutoffs for forward and reverse reads (Bowman & Arnold, 2021; Daru et al., 2018; Murray et al., 2015). Based on evaluations of the tiered mock communities, we selected forward reads for further evaluation, with a length cutoff of 240 bp and maxEE of 1.00.

Following these parameters, reads were trimmed and filtered with the ‐fastq_filter command in USEARCH (see Bowman et al., 2021). We used UNOISE3 and USEARCH to cluster sequences into Operational Taxonomic Units (OTUs) at 95% sequence similarity (UNOISE: ‐unoise3 command; USEARCH: ‐cluster_smallmem; see Bowman et al., 2021; Daru et al., 2018). Reads from negative controls were treated as above and filtered from the remaining OTUs.

After filtering as above, the data set from the even mock community comprised 494,112 reads, and the data set from the tiered mock community comprised 390,333 reads. Following our initial quality control steps, 77 OTUs were detected in the mock community data sets when OTUs were based on 95% sequence similarity, yet only 31 were expected given the DNA inputs (Table S2). We found that most of the unexpected OTUs were in the even mock community samples, and that many species that should have been detected were not, leading us to conclude that the even mock community had been contaminated and was compromised. We therefore excluded the even mock community samples, instead examining the data for the tiered mock community. We found that by removing OTUs contributing <0.15% of the total read number (see Oita et al., 2021), we detected 25 OTUs among 355,185 reads for the tiered mock community.

With this basis, we implemented the same rules for the endophyte data sets (cutoff 240 bp; maxEE = 1.00, retaining OTUs based on 95% sequence similarity as long as they represented ≥0.15% of total read abundance). Ultimately this resulted in removal of OTUs with <13 occurrences (total reads). Leaf and root samples that yielded <100 reads also were removed prior to statistical analyses.

The final data sets consisted of 612,335 reads and 1094 OTUs representing endophytes from leaves, and 983,644 reads and 1128 OTUs representing endophytes from roots. Analyses of the tiered mock community revealed that read number and operon number were positively correlated (Figure S1), such that we included abundance‐based metrics in subsequent analyses.

### 2.6 Phylogenetic diversity

We placed leaf‐ and root endophytes into a phylogenetic framework for Pezizomycotina with the Tree‐Based Alignment Selector Toolkit (T‐BAS) v.2.1 (Carbone et al., 2017, 2019). We focused on Pezizomycotina because 85% of OTUs in our data set were placed in this subphylum. Sequences were mapped to the Pezizomycotina v2.1 reference tree using the evolutionary placement algorithm (EPA) implemented in RAxML (see Carbone et al., 2017, 2019). We analyzed each plot separately, starting with the filtered amplicon data for leaves or roots. Data for each plot were assembled into OTUs as above. OTUs were retained for analysis if they occurred at least 13 times per plot, and with at least 100 reads per plot, in order to avoid skewing phylogenetic diversity by very rare OTUs. This conservative approach excluded some plots from analyses (2.1, 2.2, 2.3 for foliar endophytes; 2.1–2.3, 3.1, and 3.3 for root endophytes), but retained sufficient numbers of plots across the range of damage scores to examine the relationship of phylogenetic diversity and disturbance. Phylogenetic diversity was measured by UniFrac as Faith's phylogenetic diversity (Faith, 1992; Lozupone et al., 2006; Lozupone & Knight, 2005), implemented in T‐BAS with one representative sequence per OTU.

### 2.7 Statistical analyses

The purpose of our sampling design was to capture standing variation in leaf‐ and root endophyte communities as a function of forest disturbance due to Hurricane Maria. The heterogeneity of the forest required collection of different plant taxa in each plot. In turn, multiple plots per site, and multiple sites, were required to capture a range of damage levels across Carite State Forest.

Because plant species differed among study plots, our approach could confound damage score and plant identity as an explanatory factor in defining variation in endophyte communities. Therefore, we considered our sampling carefully prior to designing our statistical approaches. After the data processing and identification processes described below, our data set included collections from 23 plant genera representing 17 families. The majority of plant genera was collected once (78.3%), and 82.6% were found in only one of the three sites. In contrast, the most common families (Calophyllaceae, Fabaceae, Euphorbiaceae, and Melastomataceae) occurred in at least two sites (average = 2.25 sites) and in a mean of four plots overall (range: 3–5 plots, of nine). When the forest damage scores for each plot were considered, the mean scores for plots where these four families were found did not differ significantly (*F*_3,18_ = 0.1115, *p* = .9523), revealing that there was no robust relationship between the occurrence of the most common families and the spatial distribution of hurricane damage. The next most common families (Bignoniaceae, Lythraceae, Myrtaceae, and Urticaceae) occurred in 1.5 sites on average, and in a mean of 1.75 plots (range: 1–2). When these were considered with the four most common families, the damage scores for the plots where the eight most common families were found did not differ significantly (*F*_7,22_ = 0.2823, *p* = .9541). We therefore concluded that there was not a systemic bias in our data due to the presence of different plant taxa on different plots. This interpretation was upheld when we considered variation in endophyte richness among plant families: after variation in damage scores for plots was taken into account, there was no significant variation in endophyte richness among plant families for leaves or roots (*p* = .1382, *p* = .9215, respectively).

Even so, we chose to be conservative in our analyses by considering variation in plant taxonomy, as roughly half of the families represented in our collections were collected rarely. Even though recent studies suggest that variation in endophyte communities may be more profound spatially versus among families in well‐sampled neotropical forests (see Oita et al., 2021), we accounted for variation due to plant family identity by treating plant family as a random factor in the analyses described below. To further determine whether the presence of different plant taxa in each plot might confound our inference regarding disturbance, we repeated analyses with plant genus as a random factor, and then with plant family and genus nested within plots. Our results remained consistent in each case (data not shown). To account for underlying edaphic variation that also could confound our inferences, we took into account soil chemistry, which we summarized as the first dimension of the principal component (PCsoil) for analyses of nitrate, phosphorus, and potassium (Table S4). PCsoil described ~78% of the variation in these measures of soil chemistry (Tables S5 and S6, Figure S2).

With these provisions, we used multiple regression to consider how species richness of leaf and root endophytes differed as a function of disturbance, including plant family as a random factor, PCsoil as a main effect to capture variation in soil chemistry, and forest damage (log‐transformed) as a quadratic term to permit a curvilinear fit per the intermediate disturbance hypothesis. We used the same approach for analyses of phylogenetic diversity values.

For community composition analyses, we anticipated that sites proximate to one another might contain similar endophyte communities (see Higgins et al., 2014). Therefore, to evaluate the role of damage in defining endophyte community composition, we explicitly included geographic structure. We visualized communities by non‐metric multidimensional scaling (NMDS), quantified similarity with the Jaccard index (presence/absence) and Morisita index (abundance), and compared communities via analyses of similarity (ANOSIM), with *p*‐values from pairwise comparisons adjusted via Bonferroni correction. We used *p*‐values from analyses of the Jaccard index to generate a distinctiveness score that describes the degree to which endophyte communities in a plot differed from a regionally homogenous sample. Finally, we identified indicator OTUs via indicator species analysis with the multipatt–indicspecies package in R (De Cáceres, 2013; De Cáceres et al., 2010).

## 3 RESULTS

Endophytes associated with leaves and roots were diverse in plants sampled at Carite State Forest following Hurricane Maria. Following extensive quality control, we detected an average 121.6 OTUs of leaf endophytes (95% CI = 94.7–148.4) and 120 OTUs of root endophytes (95% CI = 103.6–136.5) per individual plant. The most prevalent taxa in both roots and leaves were Dothideomycetes, Sordariomycetes, and Eurotiomycetes (Figure S3), with indicator species referenced below.

### 3.1 Endophyte richness in leaves, but not roots, peaked at intermediate disturbance

When analyzed independent of forest damage scores, the richness of endophytes in leaves and roots did not vary solely with plant family or PC soil (Figures S4 and S5). We also found no meaningful variation in PC soil as a function of forest damage (Figure S6).

**FIGURE 2:**
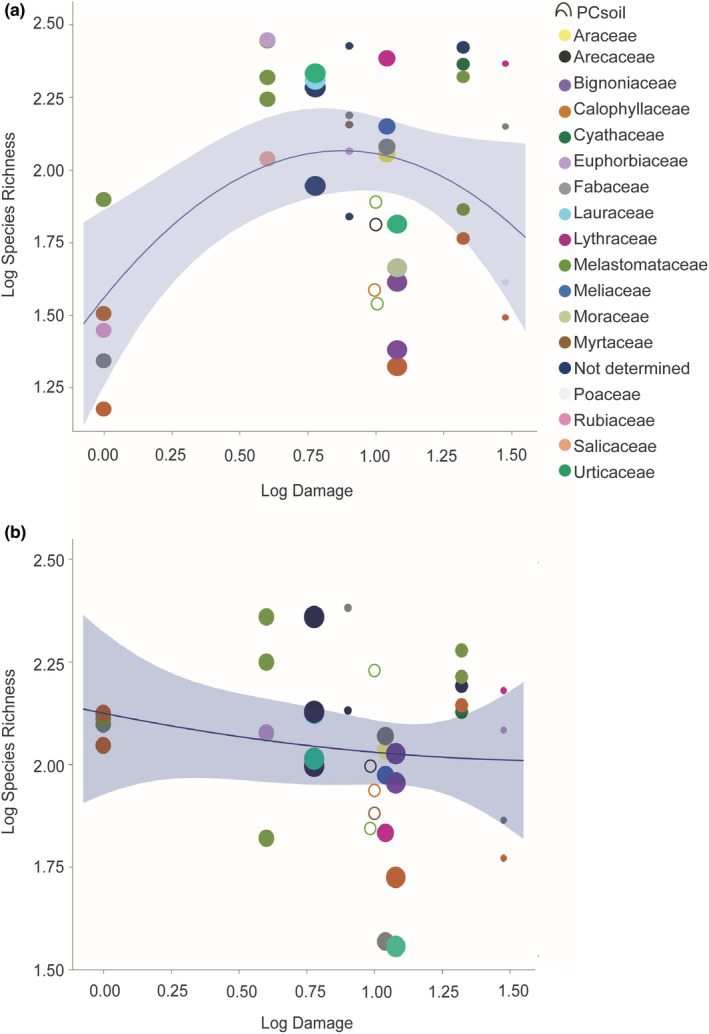
(a) Species richness of leaf endophytes varied significantly as a function of forest disturbance. Plant families are noted by color. The size of each point reflects the first principal component of soil nitrate, phosphorus, and potassium (PCsoil). Open circles represent samples with the smallest values for PCsoil, which otherwise would not be visible. Details of the multiple regression results are shown in Table 2; *R* ^*2*^ = .15, *p* = .0566). The colored band shows the 95% confidence interval around the line of fit. ND, family not determined. (b) Species richness of root endophytes did not vary as a function of forest disturbance *R* ^*2*^ = .03, *p* = .6163. Plant families Moraceae, Poaceae, Rubiaceae, and Salicaceae are not represented because individual collections were filtered during sequence editing and quality control.

However, when PC soil was taken into account and plant family was treated as a random factor, we found that endophyte richness in leaves varied as a function of forest damage scores (*R* ^2^ = .14, *p* = .0566), with a peak at intermediate disturbance levels (Table 2, Figure 2a). Endophyte richness in roots did not vary meaningfully as a function of forest damage scores (*R* ^2^ = .03, *p* = .6163; Table 2, Figure 2b). Thus data for foliar endophytes confirmed our first prediction, but endophytes belowground did not.

**TABLE 2:** Richness of leaf and root endophytes as a function of forest damage at Carite State Forest.

	Nparm	DF	DFDen	*F* ratio	Prob > *F*
Leaf endophytes					
Log damage × Log damage	1	1	28.37	11.80	.0018
PCsoil	1	1	30.94	1.40	.2464
Root endophytes					
Log damage × Log damage	1	1	29.51	0.13	.7252
PCsoil	1	1	29.84	1.12	.2991

*Note*: The analysis includes plant family as a random factor to account for differences in plant community composition among plots (see Section 2). Log‐transformed damage scores are included twice to account for the quadratic fit implied by the intermediate disturbance hypothesis.

### 3.2 Phylogenetic diversity in leaves, but not in roots, decreased at intermediate damage

**FIGURE 3:**
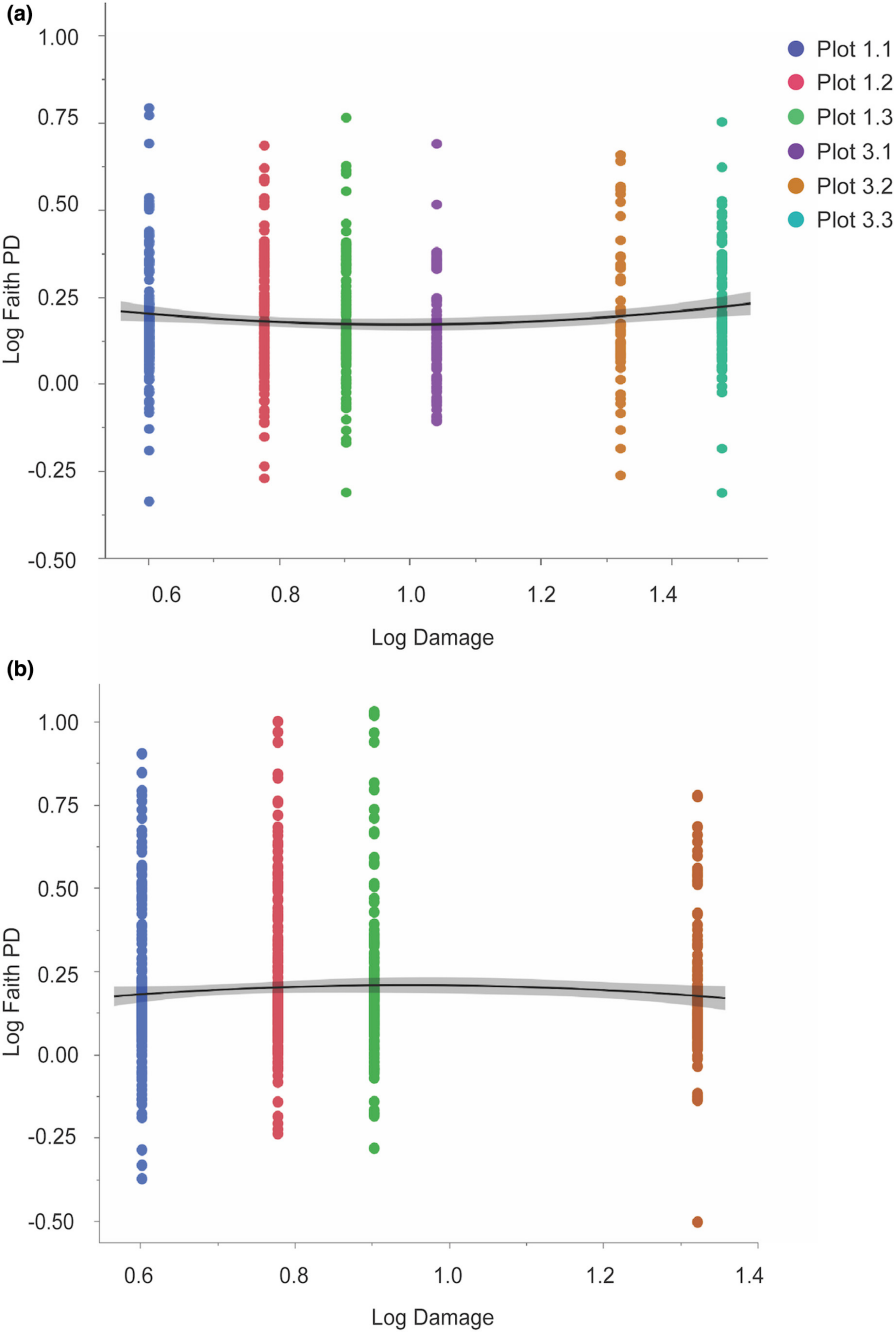
Faith's phylogenetic diversity of (a) leaf endophytes varied as a function of forest disturbance, decreasing at intermediate levels of disturbance relative to low‐ and high‐disturbance plots (quadratic fit; *p* = .0122). No such pattern was detected for (b) root endophytes (*p* = .1811). Plots not shown in the panels were excluded due to the conservative data filtering steps established for these analyses (see text).

Consistent with our second prediction, phylogenetic diversity of endophytes in leaves varied as a function of forest damage scores, with a decrease at intermediate disturbance (*F*_2,790_ = 4.428, *p* = .0122; Figure 3a). However, a similar pattern was not detected for endophytes from roots (*F*_2,864_ = 1.7120, *p* = .1811; Figure 3b). Phylogenetic diversity of endophytes in leaves (*F*_5,787_ = 1.3070, *p* = .2588) and roots (*F*_3,863_ = 0.5438, *p* = .6524) did not vary among plots when variation due to forest damage was accounted for (data not shown).

### 3.3 Endophyte communities varied spatially and as a function of forest disturbance

**FIGURE 4:**
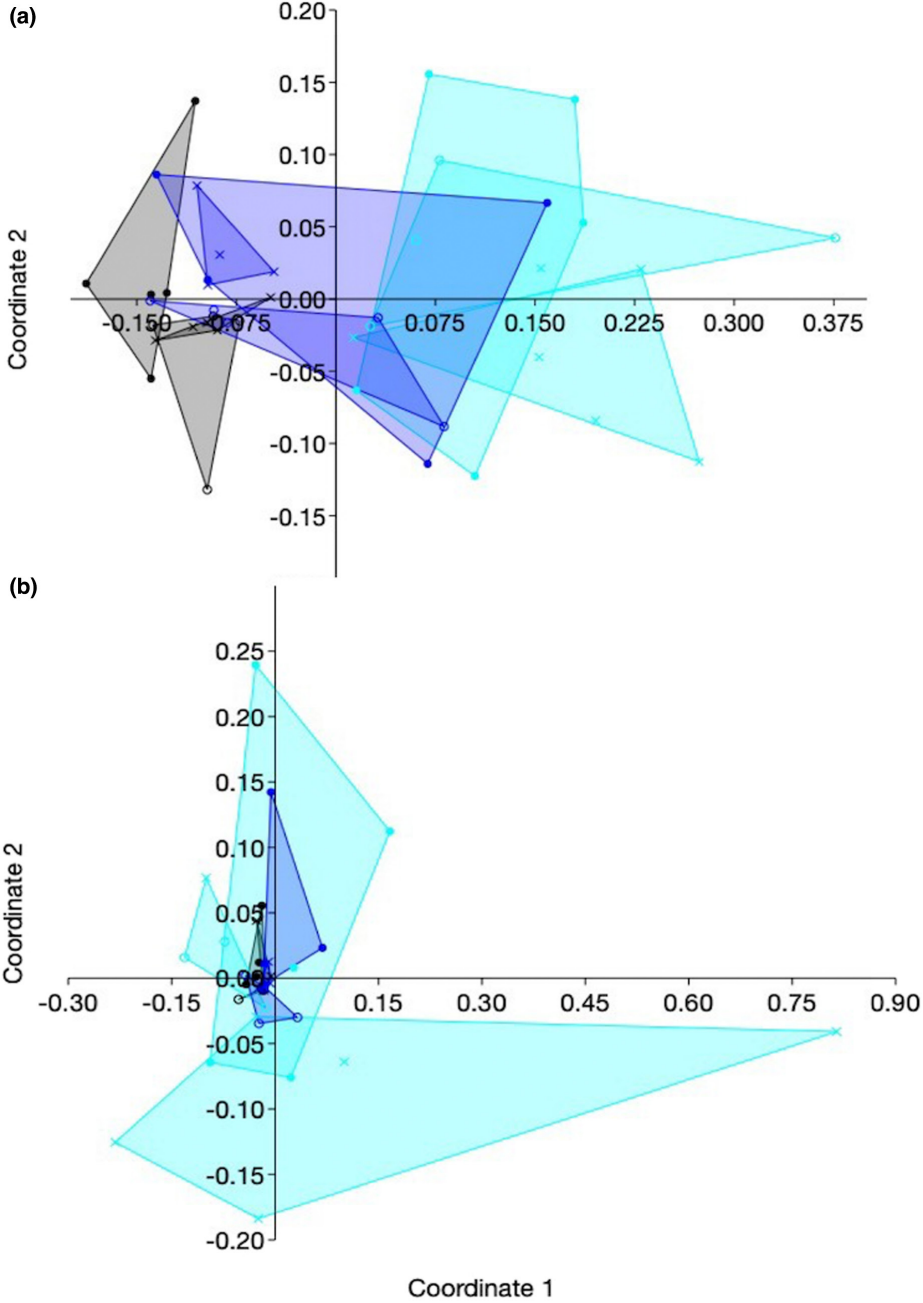
Communities of leaf endophytes differed in species composition among sites and as a function of disturbance, as illustrated by non‐metric multidimensional scaling based on (a) Jaccard index and (b) Morisita index. Symbols show local disturbance: *x*, lowest damage score within a site; open circle, intermediate damage score within a site; closed circle, highest damage score within a site. Colors: black, site 1; aqua, site 2; blue, site 3. We excluded sample 222 (site 2, plot 2, plant 2) because its endophytes were markedly different from all other samples. For statistical analyses see Table S5.

**FIGURE 5:**
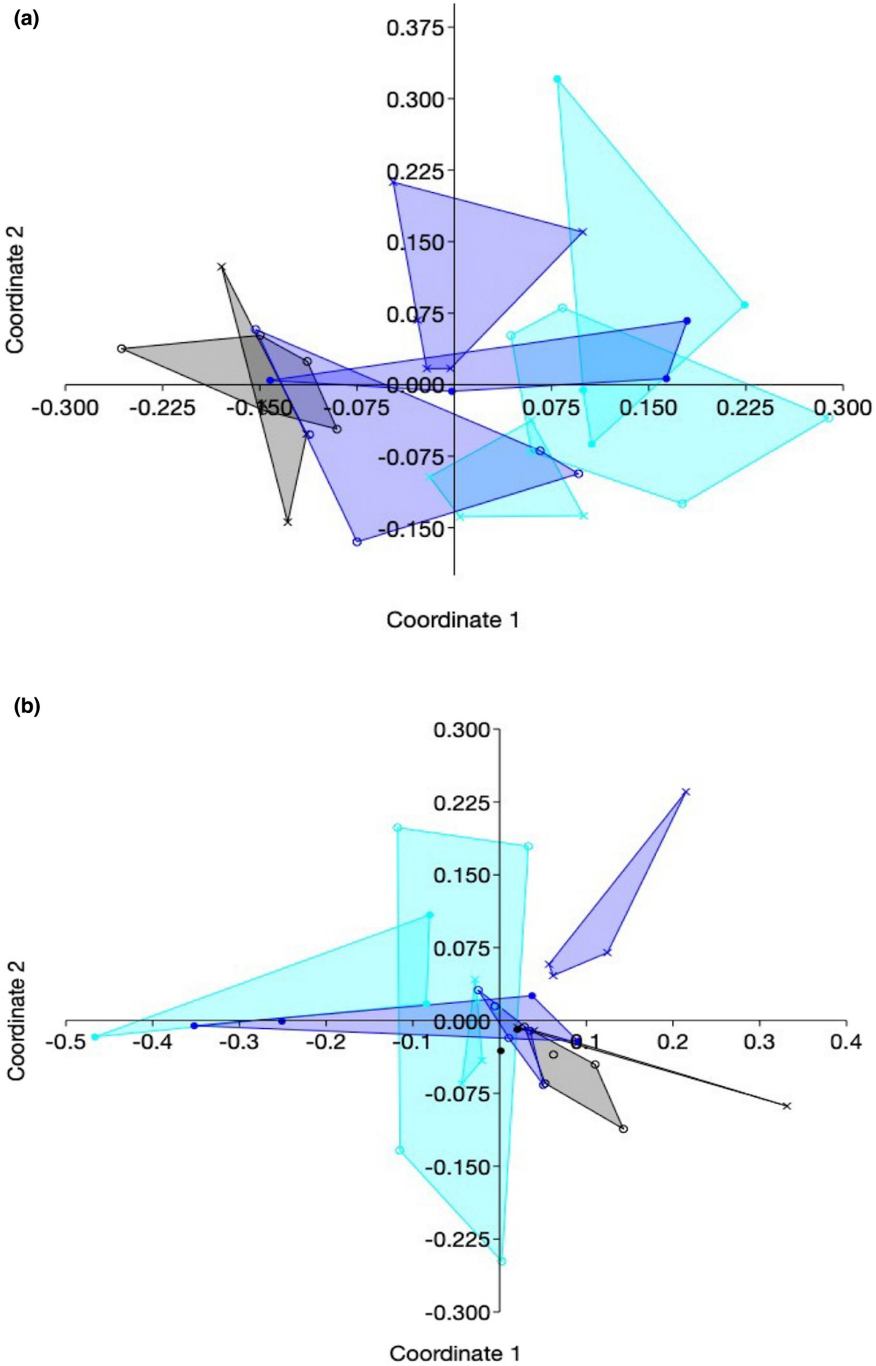
Communities of root endophytes differed in species composition among sites and as a function of disturbance, as revealed by non‐metric multidimensional scaling based on (a) Jaccard index and (b) Morisita index. Symbols show local disturbance: *x*, lowest damage score within a site; open circle, intermediate damage score within a site; closed circle, highest damage score within a site. Colors: black, site 1; aqua, site 2; blue, site 3. We excluded samples 135 and 132 (Jaccard) and 234 and 314 (Morisita; site, plot, and plant numbers as shown in Figure 4 and Table S4) because their endophytes were markedly different from all other samples. For statistical analyses see Table S5.

Sordariomycetes, Dothideomycetes, and Eurotiomycetes were the most common classes in all sites, and their prevalence was not sensitive to disturbance (Figure S3). However, as anticipated (prediction 3), species composition of endophytes in roots and leaves differed both among sites and as a function of damage scores (Figure 4: leaves, A: Jaccard index: ANOSIM: *R* = .39, *p* = .0001; B: Morisita index: ANOSIM: *R* = .11, *p* = .0008; Figure 5: roots, A: Jaccard: ANOSIM: *R* = .36, *p* = .0001; B: Morisita: ANOSIM: *R* = .21, *p* = .0003).

### 3.4 Endophyte communities in leaves, but not roots, became less distinctive in more‐disturbed areas

**FIGURE 6:**
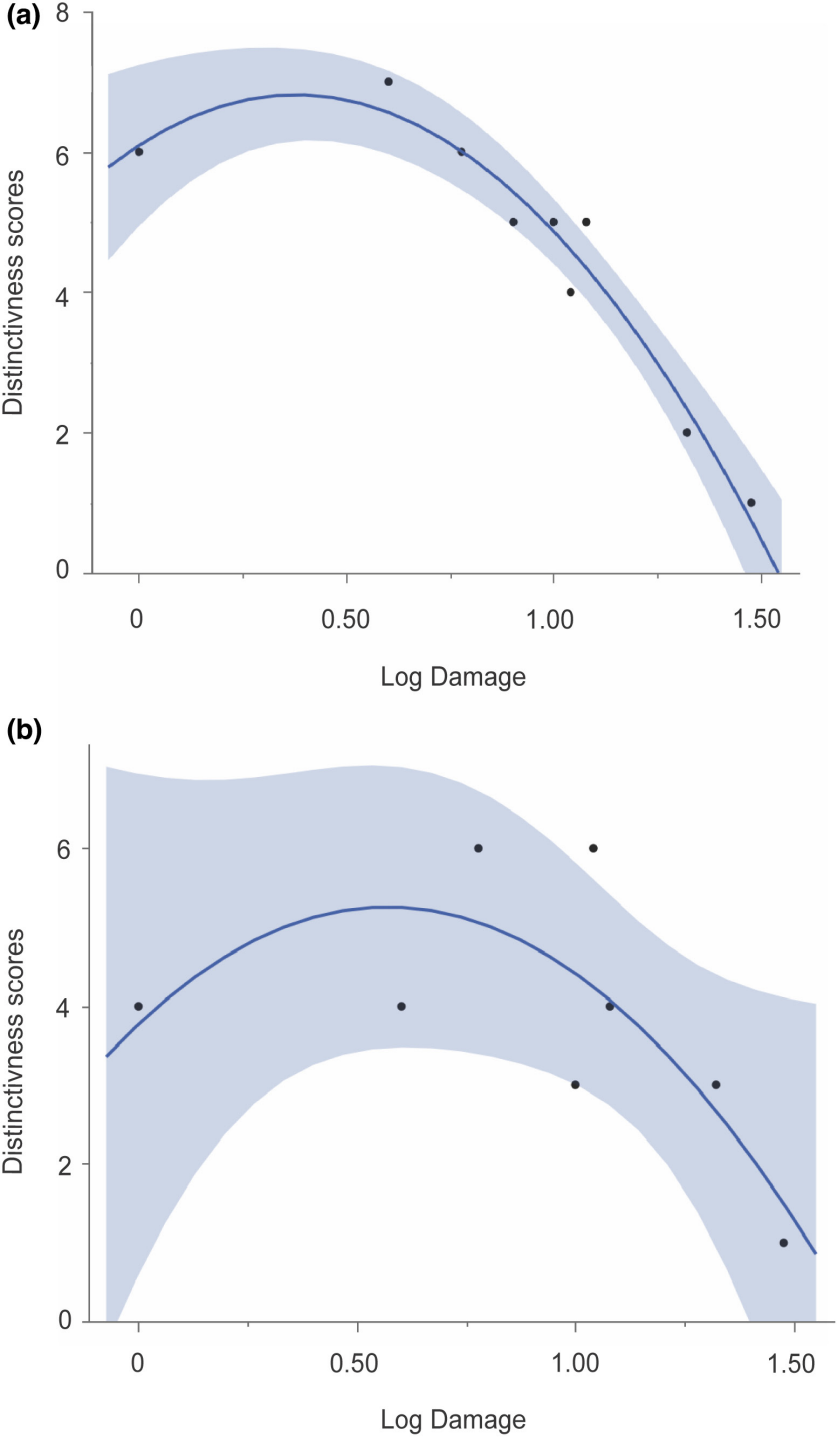
Distinctiveness score of community composition for (a) leaf endophytes and (b) root endophytes as a function of disturbance (see Table S5). The quadratic fit based on disturbance alone is significant for leaf endophytes (*R* ^*2*^ = .58; *p* < .0001) but not root endophytes (*R* ^*2*^ = .10, *p* = .1152).

As expected (prediction 4), plots with lower damage scores typically had leaf endophyte communities that were distinctive relative to those in other areas, whereas plots with more severe damage had leaf endophyte communities that were less distinctive relative to those in other sites (*R* ^2^ = .10; *p* < .0001; Table S7, Figure 6a). However, a similar pattern was not detected for endophytes from roots (*R* ^2^ = .58, *p* = .1152; Table S7, Figure 6b).

The most common leaf‐ and root endophytes differed in their prevalence as a function of forest damage (Figure S7). For leaf endophytes, 13 OTUs were identified as indicator species (Figure S7). Of these, seven were identified in plots with intermediate disturbance (*Acrodontium*, *Diplodia*, *Monilinia* (2 species), *Neodevriesia*, *Penicillium*, *Phyllosticta*), and six were identified in plots with low disturbance (*Achroiostachys*, *Aspergillus*, *Diplodia*, *Eremothecium*, *Monilinia*, *Yarrowia*). No OTUs from leaves were indicator species in high‐disturbance plots. For root endophytes, 27 OTUs were identified as indicator species (Figure S7). Three were filtered from analyses as not belonging to the Pezizomycotina. Of the remainder, five were identified in plots with high damage (*Yarrowia*, *Peziza*, *Exophiala*, *Cephalotheca*, *Rhizocarpon*), 13 in intermediate damage (*Ascobolus*, *Fusicolla*, *Cordyceps, Sarcoscypha*, *Taphrina* [3 species], *Marcelleina*, *Chrysoporthe*, *Ophiostoma*, *Curvularia*, *Stagonosporopsis*, *Urnula*), and six in low damage (*Fusarium* [2 species], *Fusicolla*, *Exophiala*, *Ophiocordyceps*, *Cyphellophora*).

## 4 DISCUSSION

Hurricanes have profound effects on forest composition and ecosystem function in the Caribbean region (Tanner et al., 1991). However, impacts of these disturbances on plant–microbe symbioses and related processes are not well understood. As hurricanes continue to increase in intensity and frequency in the Caribbean and western Atlantic (Kossin et al., 2017), cryptic effects on the symbionts with which forest trees interact may have long‐term effects on forest regeneration and resilience (see Busby et al., 2022). Charting responses of endophytes to forest disturbance due to storms like Hurricane Maria in Puerto Rico represents a first step in understanding the dynamics of the plant microbiome and the current biodiversity of endophytes in these island forests.

We found that 16 months after the passage of Hurricane Maria, communities of endophytes in forest plants in Puerto Rico showed pervasive signatures of forest disturbance due to the hurricane. Leaf‐associated endophytes peaked in richness at intermediate disturbance, but under such conditions were less phylogenetically diverse than those in areas with either little or severe forest damage. In areas with relatively less disturbance, leaf endophytes were locally distinct and their communities resembled the evolutionarily rich, mature endophyte assemblages of intact forest areas (see Arnold & Lutzoni, 2007). In areas with high disturbance, leaf endophyte communities had high phylogenetic diversity but less were distinctive relative to other local collections, consistent with perturbation and/or early succession following the hurricane's direct effects. In sites with intermediate damage, foliar endophyte communities were species‐rich but not especially phylogenetically diverse, suggesting that many closely related species may be assorting ecologically. These patterns were not observed in root endophytes, suggesting that their communities may react more slowly to disturbance, that all root endophyte communities regardless of the relative amount of disturbance had been perturbed in a relatively uniform fashion, or that spatial variation in root endophyte communities could limit our insights with regard to forest disturbance. Without samples in proximate areas without hurricane disturbance it is not clear which of these interpretations is correct, but the spatial variation observed in root endophyte community composition suggests considerable heterogeneity across the forest consistent with both damage and spatial structure. When taken together with the variation observed among leaf endophytes, a picture emerges in which heterogeneity in composition as a function of location and disturbance may translate to heterogeneity in functional traits of endophytes and endophyte–host interactions across the landscape (see Brinkmann et al., 2019; Koide et al., 2014). Exploring functional aspects of these variable associations is an important topic for future research, as it would link changes in composition of endophyte communities due to disturbance from hurricanes to the dynamics of plant communities in damaged and recovering forests.

More broadly, our study suggests that to understand impacts of hurricanes on forest processes mediated by endophytes, the appropriate sampling periods or scope may differ for tissues above and belowground. By the time of our study, leaves had generally flushed once or twice since the passage of Hurricane Maria, but the dynamics of root growth or death were not known. We anticipated that leaves would be sensitive to disturbance more immediately, and that roots could experience disturbance over longer time scales than that studied here (see Kandalepas, 2012). Even so, the differences we observed in community composition of both leaf and root endophytes are consistent with meaningful impacts by this hurricane on symbiont communities in both compartments. Our study thus provides a baseline for surveys that may detect additional changes in endophyte communities as this forest recovers from Hurricane Maria, and a basis for monitoring change following future disturbance events.

As our study was only retrospective, it is possible that variation in disturbance among our plots could reflect topography and related factors, and thus the differences we observed among endophytes could be signals of factors other than damage per se. To overcome this issue, we surveyed plots located in three sites, each encompassing a local damage gradient. Further, we accounted for differences in soil chemistry and addressed potential variation due to the presence of different plants among plots. Overall, we attribute the observed relationships of endophyte richness, phylogenetic diversity, and distinctiveness (leaves) and community composition (leaves and roots) to damage from the hurricane, but we encourage broader sampling to capture background variation in endophyte composition across the landscape. We note that the major groups of endophytes found in sites with low‐ and intermediate damage, including the most common genera, resemble those investigated by Lodge et al. (1996) in *Manilkara bidentata* of Puerto Rico, suggesting that our study captured representative endophyte communities for the region.

The findings of our study align with those of Kandalepas (2012), in which foliar endophytes in wetland plants were affected strongly by a simulated hurricane. In that study, hurricane winds acted as an agent of dispersal, homogenizing the endophyte community in the sampled plants (Kandalepas, 2012). More broadly, winds often lead to canopy opening and litter accumulation (see Cantrell et al., 2014; Everham & Brokaw, 1996; González et al., 2014; Lugo, 2000; Shiels et al., 2010; Shiels & Gonzalez, 2014; Xi & Peet, 2011; Zimmerman et al., 1996). Damage to the forest canopy can increase light intensity and temperature, change humidity and soil moisture, and, through litter accumulation, change nutrient dynamics that shape fungal biomass (Bellingham et al., 1996; Denslow et al., 1998; Fernandez & Fetcher, 1991; González et al., 2014; Harris, 1981; Landesman & Dighton, 2011; Lodge et al., 1991; Lodge et al., 2008; Lodge et al., 2022; Lugo, 2008; Mannaa & Kim, 2018; Miller & Lodge, 1997; Richardson et al., 2010; Rousk & Bååth, 2011; Turton, 1992). Increases in solar radiation and litter drying as a result of hurricane disturbance can result in the replacement of less tolerant fungal species by disturbance‐adapted species and the occurrence of homogenous fungal communities, as observed following Hurricane Hugo in Puerto Rico (Lodge & Cantrell, 1995; Vandermeer et al., 2000). We suggest that these factors may partly explain alterations in the endophyte communities observed in our findings in response to hurricane disturbance. Nonetheless, Hubbell et al. (1999) demonstrated that disturbance events would not necessarily lead to the replacement of less‐tolerant species by disturbance‐adapted species, but instead by species that are simply abundant enough at the right place and time to establish opportunitistically. Hence, we cannot rule out that the spatial structure of fungal endophytes following a major disturbance event will depend on species abundance and recovery in the region, rather than the effect of stress alone.

Overall, our results show that endophytes are sensitive to damage due to major hurricanes, and that such sensitivity can be detected well after a year post‐disturbance, particularly in foliar endophyte communities. We anticipate that widespread and repeated disturbance consistent with the increasing impact of hurricanes could lead to local extirpation of distinctive endophyte species that may have co‐evolved with key components of island floras, and which may be important for the resilience of island ecosystems. Such effects may be relatively immediate in leaves and relatively slow in roots, a prediction that could be evaluated by resampling our plots in subsequent years. Overall, our work contributes to understanding how symbiont communities that can influence plant growth and resilience are sensitive to major abiotic disturbances, which is important for predicting their function in plants under an altered climate and developing strategies to restore severely damaged ecosystems in diverse regions of the world.

## AUTHOR CONTRIBUTIONS

**Nicole Colón Carrión:** Conceptualization (supporting); data curation (lead); formal analysis (equal); funding acquisition (lead); investigation (equal); methodology (equal); project administration (supporting); visualization (supporting); writing – original draft (equal); writing – review and editing (supporting). **Chad Lozada Troche:** Project administration (supporting); resources (supporting); writing – review and editing (supporting). **A. Elizabeth Arnold:** Conceptualization (equal); data curation (supporting); formal analysis (equal); funding acquisition (equal); investigation (equal); methodology (equal); project administration (lead); resources (equal); software (equal); supervision (lead); validation (lead); visualization (equal); writing – original draft (equal); writing – review and editing (lead).

## CONFLICT OF INTEREST

No potential conflict of interest was identified by the authors.

### OPEN RESEARCH BADGES

This article has earned an Open Data badge for making publicly available the digitally‐shareable data necessary to reproduce the reported results. The data is available at [Github at https://github.com/ncoloncarrion/CariteEndophyteStudy. Amplicon sequences for plants are accessible via GenBank under accessions OP390837‐OP390867].

## Supporting information


Appendix S1.

## Data Availability

The data that support the findings of this study are openly available in Github at https://github.com/ncoloncarrion/CariteEndophyteStudy. Amplicon sequences for plants are accessible via GenBank under accessions OP390837‐OP390867.
