# Supplementary material for: Communities of endophytic fungi in a Puerto Rican rainforest vary along a gradient of disturbance due to Hurricane Maria
Source: Ecol Evol. 2022 Dec 14;12(12):e9618. doi: 10.1002/ece3.9618 (PMC9750846; doi:10.1002/ece3.9618)
Supplement: Supplementary file 1 — Appendix S1. [file ECE3-12-e9618-s001.pdf]

SUPPORTING INFORMATION

Communities of endophytic fungi in a Puerto Rican rainforest vary along a gradient of disturbance due to Hurricane Maria

Nicole Colón Carrión[1*], Chad Lozada Troche[2], A. Elizabeth Arnold[1,3]

Table S1. Measurements of stem richness and density, canopy height, diameter at breast height (DBH) of the focal tree, canopy cover, and soil pH at plots in Carite State Forest, Puerto Rico, 16 months after the passage of Hurricane Maria. Methods followed Oita et al. (2021): stem density was defined as the total number of stems with >1 cm DBH, stem richness was defined as the number of species within a 4x5 plot (≥1 cm DBH), and canopy cover was measured with a densitometer at nine points (the center of each plot, the corners, and midpoint of each plot boundary), with the score indicating the percent of those nine points with a closed canopy.

Site	Plot	Stem density (count)	Stem richness (count)	DBH (cm)	Canopy height (m)	Canopy cover (%)	Soil pH
1	1	1	1	11	5.0	44.4	7.0
1	2	2	2	2	3.0	33.3	7.0
1	3	1	1	33	2.0	44.4	7.0
2	1	1	1	30	7.6	77.8	7.0
2	2	4	2	43	1.5	77.8	7.5
2	3	3	2	14	3.0	44.4	7.0
3	1	2	1	76	3.7	55.6	6.5
3	2	1	1	29	1.8	66.7	6.5
3	3	1	1	160	4.0	33.3	6.5

Table S2. Mock community information, including taxonomic assignment and DNA concentrations of species for tiered and even mock communities, following Daru et al. (2018). ND, not determined.

Isolate	Phylum	Class or Subphylum	Order	Species	Concentration (ng/µl)	
					Tiered	Even
AZ0245	Ascomycota	Pezizomycetes	Pezizales	ND	13.00	1.84
CBS 100462	Ascomycota	Dothideomycetes	Capnodiales	*Cladosporium sphaerospermum*	3.08	1.84
CBS 107.57	Basidiomycota	Agaricomycetes	Boletales	*Suillus brevipes*	18.30	1.84
CBS 107930	Basidiomycota	Agaricomycetes	Russulales	*Hericium abietis*	2.02	1.84
CBS 110123	Ascomycota	Sordariomycetes	Hypocreales	*Lecanicillium muscarium*	23.20	1.84
CBS 116499	Ascomycota	Sordariomycetes	Amphisphaeriales	*Seimatosporium lonicerae*	11.80	1.84
CBS 119120	Ascomycota	Sordariomycetes	Hypocreales	*Trichoderma theobromicola*	2.10	1.84
CBS 121445	Ascomycota	Sordariomycetes	Sordariales	*Coniochaeta prunicola*	10.40	1.84
CBS 122753	Ascomycota	Eurotiomycetes	Phaeomoniellales	*Neophaeomoniella zymoides*	6.08	1.84
CBS 124948	Ascomycota	Sordariomycetes	Glomerellales	*Colletotrichum tropicale*	2.53	1.84
CBS 130337	Ascomycota	Sordariomycetes	Hypocreales	*Fusarium torulosum*	2.98	1.84
CBS 130859	Ascomycota	Sordariomycetes	Hypocreales	*Hypocrea orientalis*	5.44	1.84
CBS 133434	Zoopagomycota	Entomophthoromycetes	Entomophthorales	*Conidiobolus*	7.16	1.84
CBS 158.61	Ascomycota	Sordariomycetes	Hypocreales	*Acremonium kiliense*	10.20	1.84
CBS 161.73	Ascomycota	Dothideomycetes	Pleosporales	*Epicoccum nigrum*	1.84	1.84
CBS 186.71	Ascomycota	Sordariomycetes	Amphisphaeriales	*Pestalotiopsis chamaeropis*	6.48	1.84
CBS 197.82	Ascomycota	Dothideomycetes	Pleosporales	*Dendrothyrium variisporum*	10.40	1.84
CBS 210.32	Mucoromycota	Mucoromycotina	Mortierellales	*Mortierella alpina*	6.20	1.84
CBS 218.59	Zoopagomycota	Dimargaritomycetes	Dimargaritales	*Dimargaris bacillispora*	4.44	1.84
CBS 251.37	Basidiomycota	Agaricomycetes	Agaricales	*Panaeolina foenisecii*	23.60	1.84
CBS 352	Ascomycota	Schizosaccharomycetes	Schizosaccharomycetales	*Schizosaccharomyces pombe*	5.68	1.84
CBS 371.53	Basidiomycota	Ustilaginomycetes	Ustilaginales	*Ustilago cynodontis*	3.57	1.84
CBS 385.35	Ascomycota	Sordariomycetes	Xylariales	*Xylaria mali*	5.16	1.84
CBS 385.95	Mucoromycota	Mucoromycotina	Mucorales	*Mucor endophyticus*	2.30	1.84
CBS 388.52	Ascomycota	Leotiomycetes	Helotiales	*Botryotinia sp.*	5.40	1.84
CBS 408.71	Basidiomycota	Agaricomycetes	Polyporales	*Sparassis crispa*	2.08	1.84
CBS 6001	Basidiomycota	Exobasidiomycetes	Malasseziales	*Malassezia*	4.04	1.84

CBS 675.73	Chytridiomycota	Chytridiomycetes	Chytridiales	*Chytridium confervae*	13.20	1.84
CBS 6965	Basidiomycota	Tremellomycetes	Tremellales	*Tremella aurantia*	4.28	1.84
CBS 7744	Ascomycota	Saccharomycetes	Saccharomycetales	*Metschnikowia agaves*	10.60	1.84
P3112	Basidiomycota	Pucciniomycetes	ND	ND	0.94	-
Peachpath1	Ascomycota	Dothideomycetes	Pleosporales	*Phoma sp. A cf. aliena*	2.58	1.84

Table S3. Primer information for host plant identification using genomic DNA obtained from leaves, including primer name, targeted region, sequence, plant samples amplified with each, and source. Reference for all primer pairs: White et al. (1990) and Cheng et al. (2016). Plant samples 111, 113, 114, 115, 131, 135, 211, 212, 214, 221, 224, 232, 233, 234, 311, 313, 321, 322, 333, and 335 were amplified with ITS5/ITS4. Plant samples 112, 124, 133, 223, 225, 235, 315, 325, 331, and 334 were amplified by ITS-P3/ITS-U4. Plant sample 325 was amplified by ITS-P5/ITS-P3/ITS-U4.

Primers	Targeted region	Sequence (5'-3')	Amplification
ITS5/ITS4	ITS5: 18S - Forward	ITS5: GGAAGTAAAAGTCGTAACAAGG	Single
	ITS4: 26S - Reverse	ITS4: TCCTCCGCTTATTGATATGC	
ITS-P3/ITS-U4	ITS-P3: 5.8S - Forward	ITS-P3: YGACTCTCGGCAACGGATA	Single
	ITS-U4: 26S - Reverse	ITS-U4: RGTTTCTTTTCCTCCGCTTA	
ITS-P5/ITS-P3/ITS-U4	ITS-P5: 18S - Forward	ITS-P5: CCTTATCAYTTAGAGGAAGGAG	Nested
	ITS-P3: 5.8S - Forward	ITS-P3: YGACTCTCGGCAACGGATA	
	ITS-U4: 26S - Reverse	ITS-U4: RGTTTCTTTTCCTCCGCTTA	

Table S4. Plant identity and endophyte richness (OTUs) for the leaf- and root endophyte data sets collected at Carite, Puerto Rico. PCsoil: variation in soil chemistry (nitrate, phosphorus, and potassium) captured via a principal component analysis (first dimension; see Tables S5 and S6). Measurements of soil P for site 2.2 were not detected by the instrument and are listed as not determined (ND) (see Table 1). Plant samples excluded based on the filtering and quality control process for the amplicon sequence data are marked NA (not applicable; see section 2.5). As a result, the data frame used for the principal component analysis differed slightly between leaves and roots, leading to minor differences in PCsoil between the data sets. Plants that could not be identified due to degradation of material are shown as ND.

Site	Plot	Sample	Plant family	Plant genus	Endophyte richness		PCsoil	
					Leaves	Roots	Leaves	Roots
1	1	1	Euphorbiaceae	*Alchornea*	280	119	0.462	0.365
1	1	2	Salicaceae	*Casearia*	109	NA	0.462	NA
1	1	3	Melastomataceae	*Clidemia*	278	66	0.462	0.365
1	1	4	Melastomataceae	*Miconia*	208	177	0.462	0.365
1	1	5	Melastomataceae	*Nepsera*	175	288	0.462	0.365
1	2	1	ND	ND	NA	228	NA	2.208
1	2	2	Urticaceae	*Pourouma*	215	103	2.365	2.208
1	2	3	ND	ND	88	99	2.365	2.208
1	2	4	Lauraceae	*Ocotea*	204	133	2.365	2.208
1	2	5	ND	ND	192	134	2.365	2.208
1	3	1	Euphorbiaceae	*Alchornea*	116	NA	-1.650	NA
1	3	2	ND	ND	267	135	-1.650	-1.710
1	3	3	Myrtaceae	*Syzygium*	143	NA	-1.650	NA
1	3	4	ND	ND	69	NA	-1.650	NA
1	3	5	Fabaceae	*Inga*	154	240	-1.650	-1.710

2	1	1	Melastomataceae	*Miconia*	79	129	0.325	0.232
2	1	2	Calophyllaceae	*Calophyllum*	32	133	0.325	0.232
2	1	3	Fabaceae	*Pterocarpus*	22	125	0.325	0.232
2	1	4	Rubiaceae	*Gonzalagunia*	28	NA	0.325	NA
2	1	5	Calophyllaceae	*Calophyllum*	15	111	0.325	0.232
2	2	1	Calophyllaceae	*Calophyllum*	37	88	ND	ND
2	2	2	Myrtaceae	*Eucalyptus*	NA	77	ND	ND
2	2	3	Arecaceae	*Adonidia*	65	97	ND	ND
2	2	4	Melastomataceae	*Miconia*	79	174	ND	ND
2	2	5	Melastomataceae	*Miconia*	36	71	ND	ND
2	3	1	Moraceae	*Ficus*	46	NA	1.993	NA
2	3	2	Bignoniaceae	*Amphitecna*	41	106	1.993	1.847
2	3	3	Bignoniaceae	*Handroanthus*	24	90	1.993	1.847
2	3	4	Urticaceae	*Cecropia*	65	36	1.993	1.847
2	3	5	Calophyllaceae	*Calophyllum*	21	53	1.993	1.847
3	1	1	Fabaceae	*Inga*	120	117	0.844	0.766
3	1	2	Meliaceae	*Guarea*	141	94	0.844	0.766
3	1	3	Lythraceae	*Lagerstroemia*	242	68	0.844	0.766
3	1	4	*Fabaceae*	*Pterocarpus*	NA	37	NA	0.766
3	1	5	Araceae	Araceae	113	108	0.844	0.766
3	2	1	Melastomataceae	*Miconia*	209	189	-0.461	-0.524
3	2	2	Melastomataceae	*Miconia*	73	163	-0.461	-0.524
3	2	3	ND	ND	264	155	-0.461	-0.524
3	2	4	Calophyllaceae	*Calophyllum*	58	139	-0.461	-0.524
3	2	5	Cyathaceae	*Cyathea*	231	134	-0.461	-0.524
3	3	1	Poaceae	*Sorghum*	41	NA	-1.835	NA
3	3	2	Lythraceae	*Lagerstroemia*	232	151	-1.835	-1.862
3	3	3	Euphorbiaceae	*Alchornea*	141	73	-1.835	-1.862
3	3	4	Calophyllaceae	*Calophyllum*	31	59	-1.835	-1.862
3	3	5	*Euphorbiaceae*	*Alchornea*	NA	121	NA	-1.862

Table S5. Eigenvectors of the first three dimensions of the principal component analysis of soil chemistry (nitrate, phosphorus, and potassium).

	First dimension	Second dimension	Third dimension
ppm NO_3^-	-0.60	0.43	0.68
ppm P	0.60	-0.32	0.73
ppm K	0.53	0.85	-0.06

Table S6. Correlation matrix of principal component analysis (first dimension, PCsoil) of soil chemistry variables (potassium, nitrate, and phosphorus) estimated by restricted maximum likelihood (REML).

	ppm NO_3-	ppm P
ppm NO_3-		
ppm P	-0.81	
ppm K	-0.57	0.60

Table S7. Pairwise comparisons among plots of leaf endophyte communities (A) and root endophyte communities (B). Values were obtained from ANOSIM based on Jaccard index (below diagonal, based on presence-absence data) and Morisita index (above diagonal, based on abundance data), adjusted via Bonferroni correction. Significant values indicate significantly different species composition. Distinctiveness scores are calculated as the number of times a plot is significantly different in endophyte species composition relative to others. NA = not included due to limitations with the data; see Methods.

Leaf endophytes									
Plot	1.1	1.2	1.3	2.1	2.2	2.3	3.1	3.2	3.3
1.1		0.1284	0.0302	0.00092	0.0092	0.01	0.0282	0.6063	0.0254
1.2	0.0497		0.3863	0.4778	0.0303	0.8251	0.3728	0.3347	0.1396
1.3	0.0074	0.0322		0.0085	0.0086	0.0083	0.2412	0.1914	0.1607
2.1	0.0075	0.0138	0.0067		0.4152	0.094	0.3439	0.0569	0.6374
2.2	0.0081	0.0277	0.0098	0.024		0.8456	0.0573	0.0984	0.6503
2.3	0.008	0.0175	0.0086	0.1115	0.168		0.7846	0.0078	0.986
3.1	0.0156	0.0316	0.0725	0.0165	0.028	0.0245		0.4117	0.4639
3.2	0.4565	0.3802	0.0729	0.0223	0.0919	0.0171	0.1629		0.1108
3.3	0.014	0.0613	0.064	0.0821	0.1231	0.1961	0.2298	0.2271	
Root endophytes									
1.1		0.1688	0.5345	0.0305	0.2855	0.0612	0.0277	0.3406	0.2023
1.2	0.0806		0.2807	0.0085	0.0074	0.0178	0.0093	0.0694	0.0261
1.3	NA	NA		0.5379	0.9044	0.5064	0.3362	0.6099	0.528
2.1	0.0266	0.0091	NA		0.3561	0.0602	0.0297	0.2464	0.0537
2.2	0.0086	0.0082	NA	0.1937		0.5675	0.0331	0.0098	0.2762
2.3	0.0275	0.0085	NA	0.0549	0.4788		0.0281	0.0164	0.4932
3.1	0.0079	0.0077	NA	0.0109	0.0066	0.0076		0.0088	0.1706
3.2	0.4284	0.039	NA	0.5434	0.1352	0.0381	0.0076		0.034
3.3	0.1688	0.0159	NA	0.1149	0.6724	0.6016	0.0773	0.335	

Figure S1. Positive association between read number and operon number, both ln-transformed, in the tiered mock community ($R^2 = 0.51$, $p < 0.0001$).

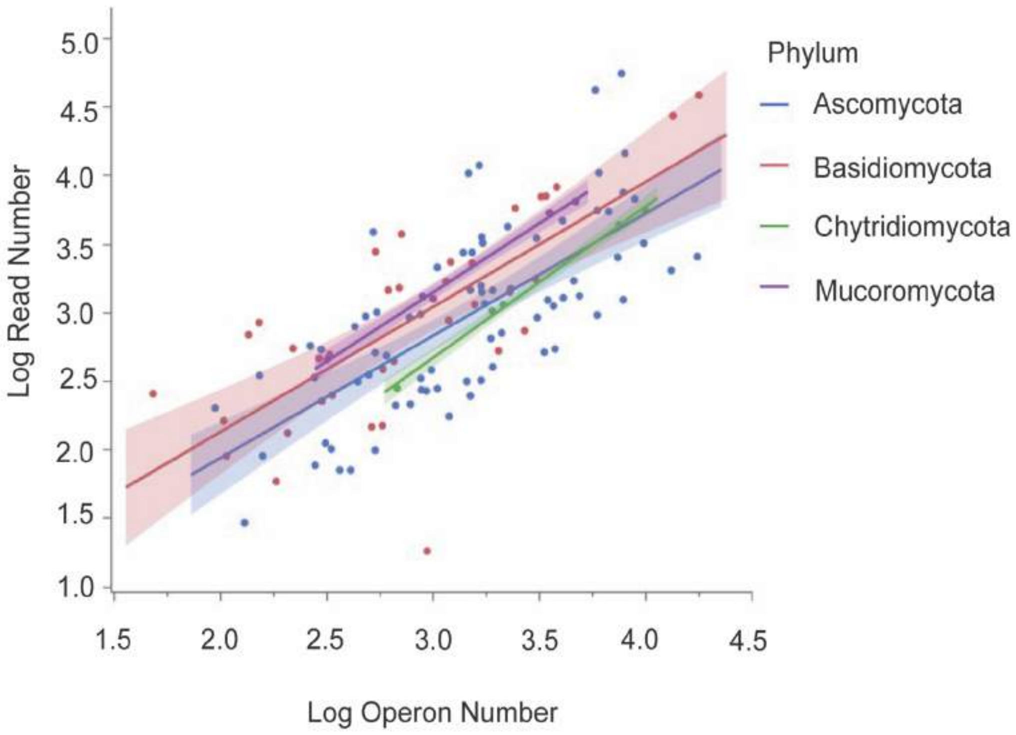

Figure S2. Principal component analysis (first dimension, PCsoil) of soil chemistry variables (nitrate, phosphorus, and potassium).

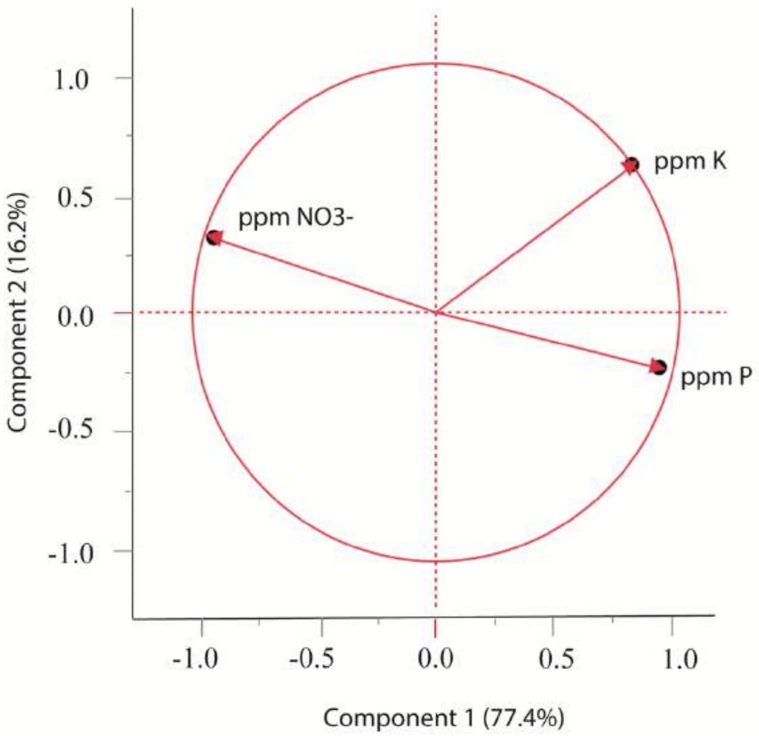

Figure S3. Most prevalent taxa among leaf endophytes at each plot (A), and as a function of disturbance (B), and root endophytes at each plot (C) and as a function of disturbance (D). Dark blue, Dothideomycetes; white-black dashes, Eurotiomycetes; green, Leotiomycetes; fuschia, Sordariomycetes; grey, other Ascomycota.

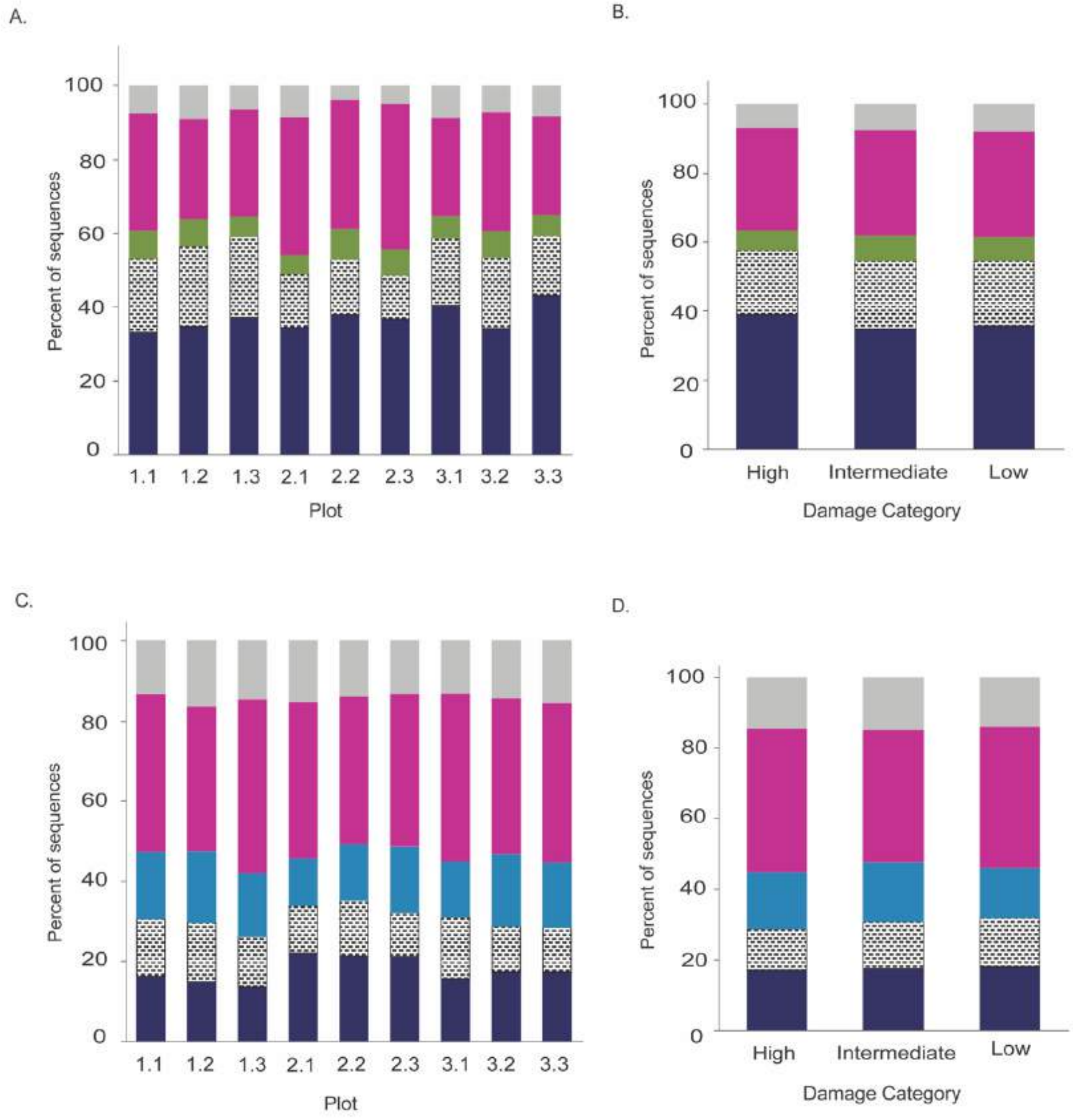

Figure S4. Endophyte richness did not vary meaningfully among plant families when damage scores were not considered. (A) Leaf endophytes, *Chi* sq = 24.76, *DF* = 17, $p = 0.1008$. (B) Root endophytes, ANOVA, $F_{13,24} = 0.6668$, $p = 0.7744$). .

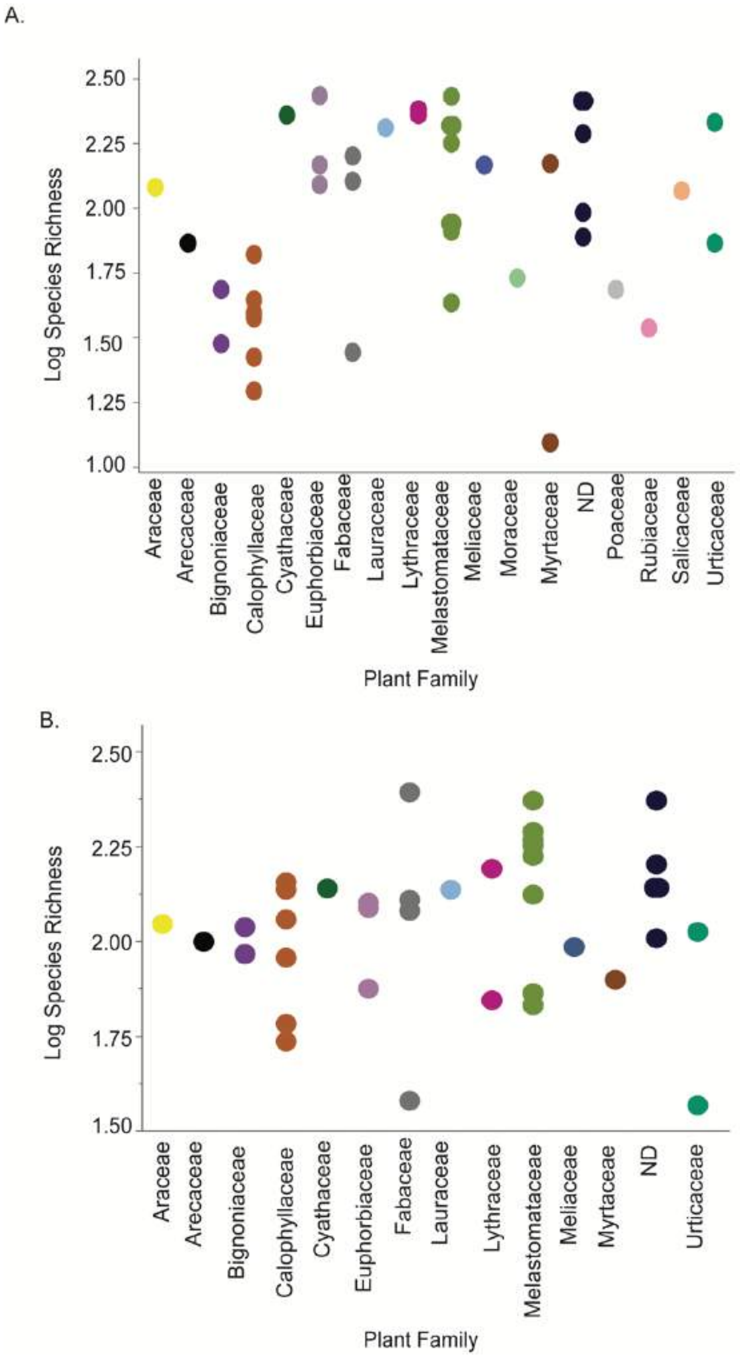
A.
Log Species Richness
2.50
2.25
2.00
1.75
1.50
1.25
1.00
Araceae
Arecaceae
Bignoniaceae
Calophyllaceae
Cyathaceae
Euphorbiaceae
Fabaceae
Lauraceae
Lythraceae
Melastomataceae
Meliaceae
Moraceae
Myrtaceae
ND
Poaceae
Rubiaceae
Salicaceae
Urticaceae
Plant Family
B.
Log Species Richness
2.50
2.25
2.00
1.75
1.50
Araceae
Arecaceae
Bignoniaceae
Calophyllaceae
Cyathaceae
Euphorbiaceae
Fabaceae
Lauraceae
Lythraceae
Melastomataceae
Meliaceae
Myrtaceae
ND
Urticaceae
Plant Family

Figure S5. Relationship between species richness of endophytes and PCsoil. (A) Leaf endophyte data frame. Linear fit based on PCsoil alone is not significant (R^2 = 0.017, p = 0.4441). (B) Root endophyte data frame. Linear fit based on PCsoil alone is not significant (R^2 = 0.044, p = 0.2407). Points are colored by plant family; ND = not determined. Grey band = 95% confidence interval.

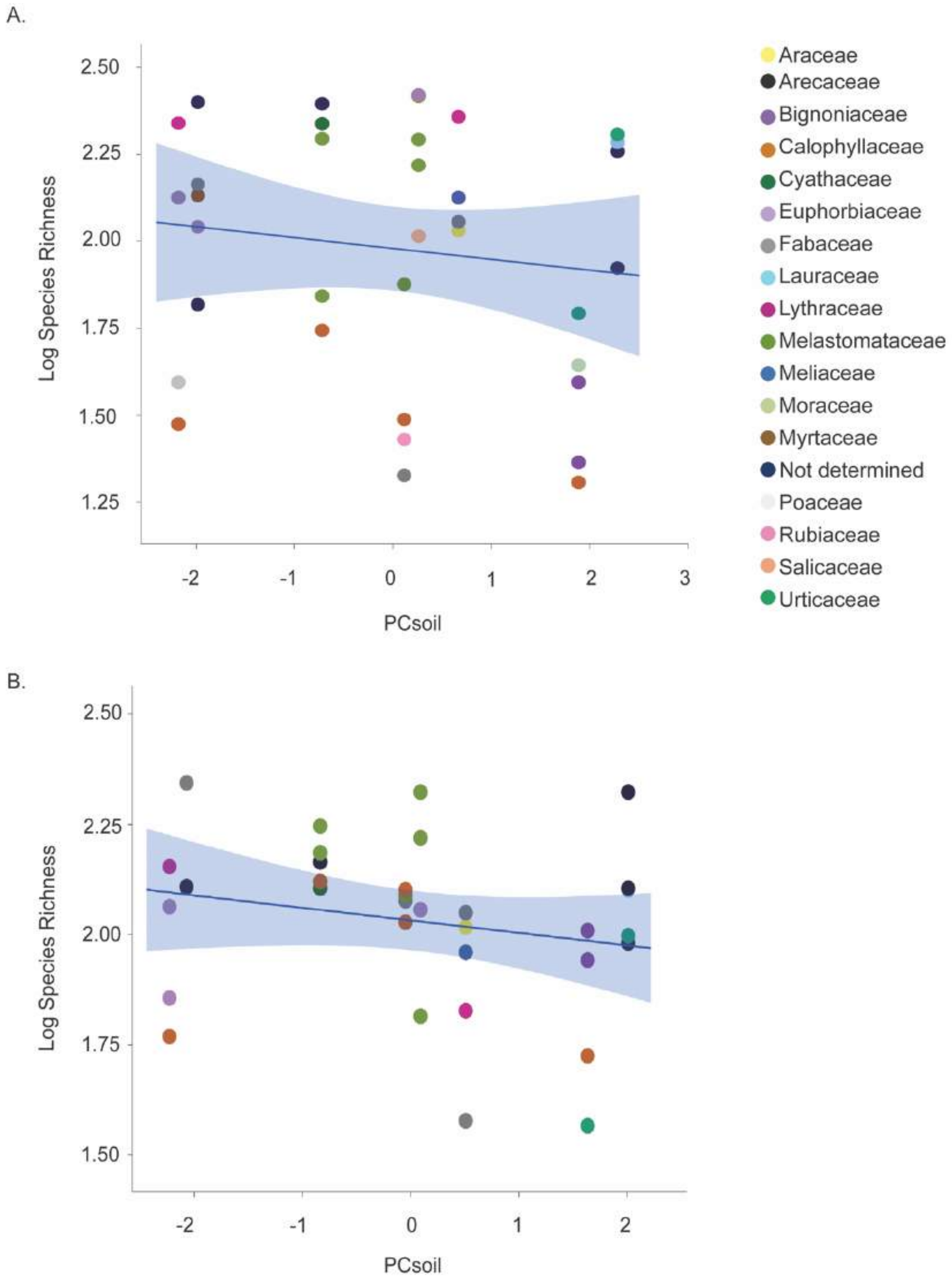
A.
2.50
2.25
2.00
1.75
1.50
1.25
Log Species Richness
-2
-1
0
1
2
3
PCsoil
Araceae
Arecaceae
Bignoniaceae
Calophyllaceae
Cyathaceae
Euphorbiaceae
Fabaceae
Lauraceae
Lythraceae
Melastomataceae
Meliaceae
Moraceae
Myrtaceae
Not determined
Poaceae
Rubiaceae
Salicaceae
Urticaceae
B.
2.50
2.25
2.00
1.75
1.50
Log Species Richness
-2
-1
0
1
2
PCsoil

Figure S6. Soil traits (PCsoil) did not vary significantly with forest damage ($R^2 = 0.073$, $DF = 1, 35$, $p = 0.1036$).

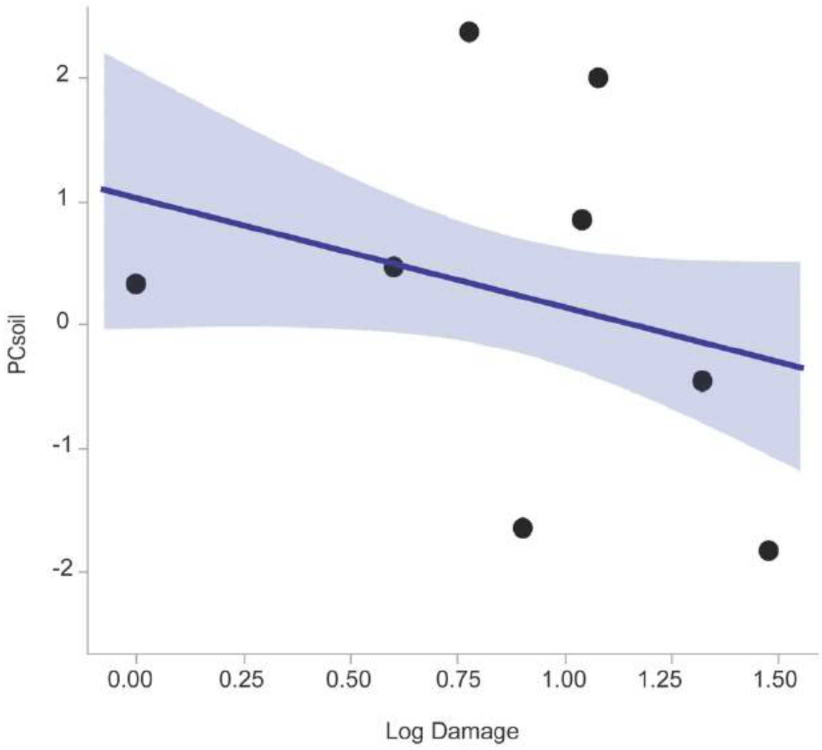

Figure S7. Indicator species as a function of damage scores for (A) leaf endophytes and (B) root endophytes. Each colored box represents an OTU identified by indicator species analysis. Colors correspond to the Indicator Value (IndVal) associated with each taxon. Indicator species were identified only to the genus level due to the short length of MiSeq sequences. Phylogenetic placement of all indicator species from leaf sequences were inferred by T-BAS. Sequences unable to be placed by T-BAS were compared via BLASTn to fungal records in GenBank. Sequences returning as "unclassified" or not belonging to the Pezizomycotina were filtered from analyses.

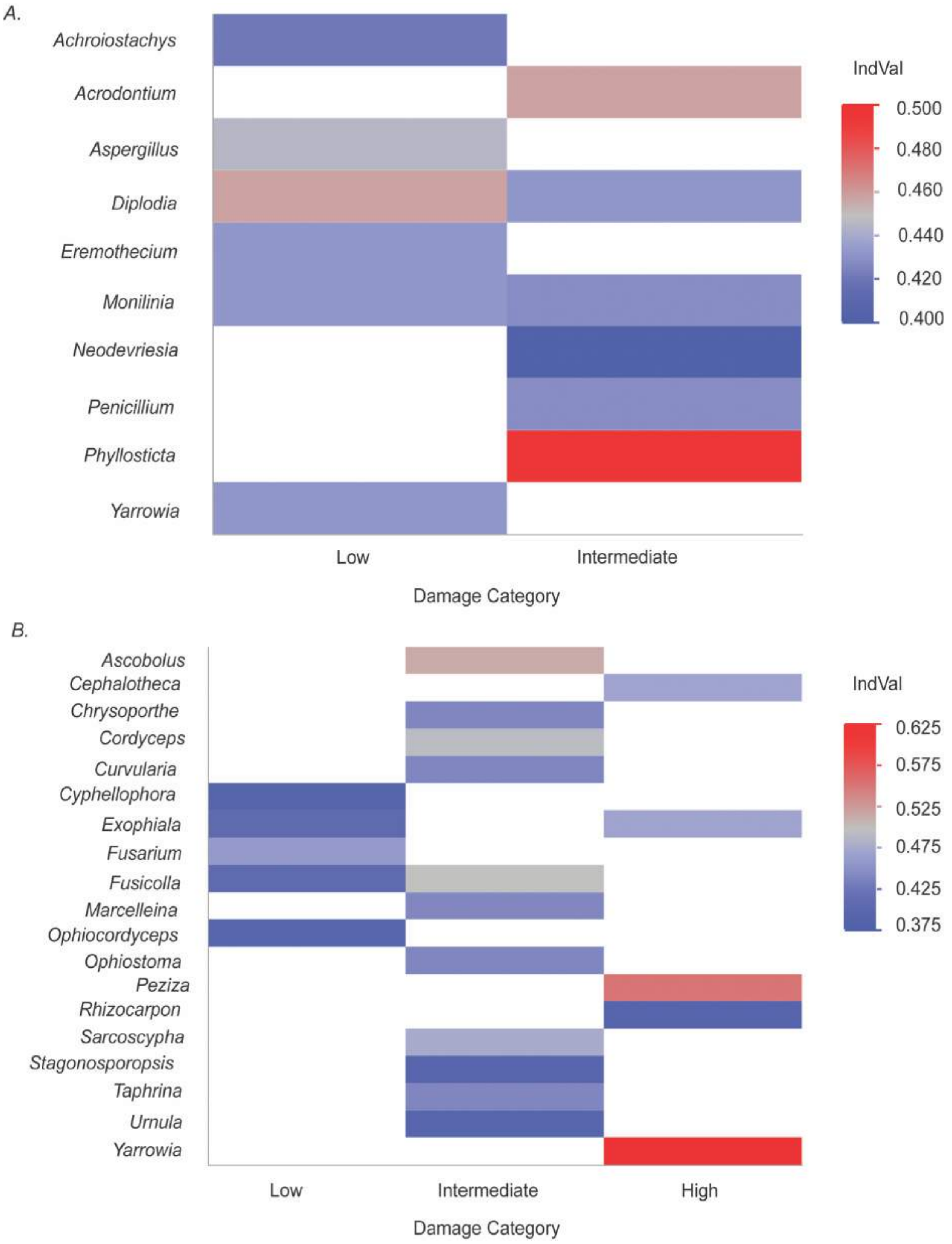
A.
Achroiostachys
Acrodontium
Aspergillus
Diplodia
Eremothecium
Monilinia
Neodevriesia
Penicillium
Phyllosticta
Yarrowia
IndVal
0.500
0.480
0.460
0.440
0.420
0.400
Low
Intermediate
Damage Category
B.
Ascobolus
Cephalotheca
Chrysoporthe
Cordyceps
Curvularia
Cyphellophora
Exophiala
Fusarium
Fusicolla
Marcelleina
Ophiocordyceps
Ophiostoma
Peziza
Rhizocarpon
Sarcoscypha
Stagonosporopsis
Taphrina
Urnula
Yarrowia
IndVal
0.625
0.575
0.525
0.475
0.425
0.375
Low
Intermediate
High
Damage Category
